# Assessment of Viral Genotype Impact to the Cost-Effectiveness and Overall Costs of Care for Peg-Interferon-2α + Ribavirine Treated Chronic Hepatitis C Patients

**DOI:** 10.5812/hepatmon.6750

**Published:** 2013-06-19

**Authors:** Mihajlo Jakovljevic, Zeljko Mijailovic, Biljana Popovska Jovicic, Predrag Canovic, Olgica Gajovic, Mirjana Jovanovic, Dejan Petrovic, Olivera Milovanovic, Natasa Djordjevic

**Affiliations:** 1 Pharmacology and Toxicology Department, The Faculty of Medical Sciences Kragujevac, University of Kragujevac, Kragujevac, Serbia; 2 Infectious Diseases Clinic, University Clinical Center Kragujevac, Kragujevac, Serbia; 3 Regional Addiction Disorders Center, Psychiatry Clinic, University Clinical Center Kragujevac, Kragujevac, Serbia; 4 Urology and Nephrology Clinic, University Clinical Center Kragujevac, Kragujevac, Serbia; 5 Department of Pharmacy The Faculty of Medical Sciences University of Kragujevac, Kragujevac, Serbia

**Keywords:** Cost-Benefit Analysis, Interferons, Ribavirin, Hepatitis C, Chronic

## Abstract

**Background:**

Pegylated interferon alfa plus ribavirin protocol is currently considered the most efficient hepatitis C treatment. However, no evidence of costs comparison among common viral genotypes has been published.

**Objectives:**

We aimed to assess core drivers of hepatitis C medical care costs and compare cost effectiveness of this treatment among patients infected by hepatitis C virus with genotypes 1 or 4 (group I), and 2 or 3 (group II).

**Patients and Materials:**

Prospective bottom-up cost-effectiveness analysis from societal perspective was conducted at Infectious Diseases Clinic, University Clinic Kragujevac, Serbia, from 2007 to 2010. There were 81 participants with hepatitis C infection, treated with peg alpha-2a interferon plus ribavirin for 48 or 24 weeks. Economic data acquired were direct inpatient medical costs, outpatient drug acquisition costs, and indirect costs calculated through human capital approach.

**Results:**

Total costs were significantly higher (P = 0.035) in group I (mean ± SD: 12,751.54 ± 5,588.06) compared to group II (mean ± SD: 10,580.57 ± 3,973.02). In addition, both direct (P = 0.039) and indirect (P < 0.001) costs separately were significantly higher in group I compared to group II. Separate comparison within direct costs revealed higher total cost of medical care (P = 0.024) in first compared to second genotype group, while the similar tendency was observed for total drug acquisition (P = 0.072).

**Conclusion:**

HCV genotypes 1 and 4 cause more severe clinical course require more care and thus incur higher expenses compared to HCV 2 and 3 genotypes. Policy makers should consider willingness to pay threshold differentially depending upon HCV viral genotype detected.

## 1. Background

Hepatitis C virus (HCV) is well known for its capability of causing long-term liver infection and consecutive inflammatory response (1). In the long run, HCV infection has been proved to substantially increase the risk of hepatic cirrhosis and hepatocellular carcinoma (2). Among several different treatment options offered in the market, pegylated interferon alfa (PEG-IFN-α) based protocol with add-on ribavirin (RBV) is currently considered the most efficient in achieving lasting remissions (3, 4). Numerous factors, including age, sex, duration of the disease, and the dosage regimen, significantly affect the outcome of the treatment (5, 6). Yet, the most important determinant of patient response to the peg-INF-2α based therapy seems to be the genotype of hepatitis C virus (3, 5-7). While efficacy and safety of PEG-INF-α plus RBV therapy have been widely investigated and supported by a significant body of evidence (8), there is a knowledge gap regarding primary cost-effectiveness data in treating patients with hepatitis infected by genotypically different HCV. Among numerous papers addressing the issue of cost-effectiveness of hepatitis C INF-based treatment, there is not a single one dealing with viral genotype as a possible core driver of medical care consumption and expenditure (9-13).

## 2. Objectives

Thus, this study aimed to determine and compare cost-effectiveness value of peg-INF-α2a plus RBV treatment of chronic hepatitis C between two prognostically different patient populations occurring in an everyday infectologist clinical practice, namely patients affected by either HCV genotypes 1 or 4, and those affected by genotypes 2 or 3 (14, 15). The trial also provided an insight into overall direct medical and indirect costs comparison between the groups, and described the key factors affecting overall expenditure.

## 3. Patients and Methods

### 3.1. Design of the Trial

This cost-effectiveness trial has been conducted alongside the clinical trial on efficiency, and it was designed as the case series study. In addition, societal perspective, as the most comprehensive one, was taken into account. Study was approved by the Ethical Committee of the Clinical Center Kragujevac, Serbia N°01/912, issued on 08.02.2010. Its registration number is GUARD-C, MV 22255 (Roche®).

### 3.2. Participants

The study enrolled patients admitted due to the chronic hepatitis C (CHC) infection to the Infectious Diseases Clinic, Clinical Centre Kragujevac, at the city of Kragujevac, Serbia, from 2007 to 2010. There were 81 patients who met all the inclusion criteria, i.e. clinically and pathohistologically confirmed active disease, the presence of detectable level of viral RNA load in plasma, as well as the willingness to participate, expressed through signed patient consent formulary. Exclusion criteria were the presence of contraindications for interferon administration, naturally occurring remission of disease, and the presence of additional severe contingent disorder unrelated to C virus routes of infection. Sample size calculation, has been performed by means of freely available software package G-Power 3.1.2 (16). We inputted probability of α type error 0.05, study power (1-β err prob) of 0.8 and used two tailed zero hypothesis testing using Student T test – Correlation: point biserial model. Thus we came to the following output: noncentrality parameter δ = 2.85, critical t = 1.99, Df =80, total sample size of 82 and actual study power of 0.80. It was calculated to ensure the detection of clinically relevant response to treatment for the level of viral RNA (decrease of 100,000 copies/ml after full course of treatment during 24-48 weeks). This choice was in line with health economic guidelines and recommendations (9). According to the presence of particular viral genotype in their blood samples, and thus the expected clinical course and treatment responses (3, 5-7), Patients with CHC were assigned to either genotype group I (infected by HCV genotypes 1 or 4), or genotype group II (having HCV genotypes 2 or 3 virus infection). In addition, small number of patients with concomitant disorders related to C virus route of infection was included in the analysis. They either had confirmed intravenous drug abuse history, or have been on a dialysis treatment of renal failure, during high-risky period of social poverty in the 90ties. Other disorders were considered insignificant for their clinical and economic consequences, either due to low frequency or due to low severity.

### 3.3. Intervention

Treatment protocol was initiated only in patients with detected viral RNA value ≥ 50 IU/mL or ≥ 40 copies/ml (all zero point values were, in fact, ≥ 140,000 copies/ml) and pathohistologically confirmed activity of necroinflammatory process and fibrosis at the liver tissue biopsy specimen. Antiviral treatment protocol included subcutaneous administration of PEG INF-α2a in a dosage range 90-180mcg once weekly, and RBV 1000-1200mg by mouth daily (depending on the body weight). There are two interferon preparations with marketing approvals In Serbia issued by The National Medicines and Medical Devices Agency *(ALIMS) indicated for treatment of chronic viral hepatitis C infection - Peginterferon alfa-2a and Peginterferon alfa-2b. In our trial we have administered Peginterferon alfa-2a (brand name „Pegasys”). The duration of the treatment, according to the current guidelines recommendations (17), was 48 weeks in genotype group I, and 24 weeks for genotype group II. In case of rapid positive response (viral RNA under detection threshold of the commercial assay) first group treatment duration was shortened to 24 weeks and the second one to only 16 weeks. The sustained virological response (SVR) as an outcome measure was considered therapeutic success (18).

### 3.4. Data Collection

Patients were observed for 24 or 48 weeks, depending on the protocol. Clinical and laboratory data were acquired by attending infectologist during regular examinations and by blood sampling, respectively, before the beginning of the intervention and at the end of the follow up. In case of sudden or unpredictable severe drug-induced adverse event, further participation of the patient was terminated. At baseline and 3, 6, 12 and 18 months after the inclusion in the study, patients were subjected to PCR determination of HCV viral response to treatment, as well as to common blood biochemistry and cytology analysis. Exact inpatient cost matrix per person treated was extracted retrospectively from large tertiary care university hospital invoice registry and by reconstruction of outpatient services consumption. Key indirect costs related to absenteeism and lost productivity were estimated based on employment status and current official average salaries in the country per education level (19). It was assumed that patients were unable to work in a week they received immunobiological treatment, and the average value of week labor wages in a given year were multiplied to calculate full lost productivity expressed in local market value. In case of random missing data, complete case analysis approach was introduced.

### 3.5. Health Outcomes

The core health outcomes observed for the cost-effectiveness analysis were based on the laboratory analysis of HCV viral RNA load. Qualitative genotypization test applied to determine HCV viral genotype in patients was: LINEAR ARRAY HCV Genotyping Test of Swiss production. Its sensitivity was 500 IU/ml. Quantitative genotypization test applied was Cobas Ampliprep / Cobas TaqMan HCV Test, US manufactured. Its sensitivity (determination limit for viral HCV RNA copies) was 15 IU/ml (result was multiplied by 2, 7 to calculate actual level of RNA copies presence) (20). Cross-sections with blood sampling were performed at baseline, then 3, 6 and 12 months after the study inclusion, and 18 months later, i.e. 6 months after cessation of the treatment. Viral genotype was determined in each patient prior to study inclusion by commercial PCR testing. Core indicator of the treatment success (achieving rapid and/or SVR) was assessed based on these outcomes (18). Liver biopsy was performed in 78 of 81 patients. FibroTest®, and liver stiffness measurement (LSM) using Fibroscan® test, was not available in local market at the time. Evaluation of liver biopsy specimen was rather qualitative, conducted by experienced pathologists. The outcome classified patients in four different stages of disease activity (1-4). Other health outcomes of interest were: the presence of risk factors for infection transmission, transaminases enzymes (ALT, AST) levels, alpha fetoprotein, conjugated and unconjugated bilirubin levels, complete blood cell count (erythrocytes, leucocytes, granulocytes, platelets), viral RNA load, blood proteins, albumins, globulins, INR (international normalized ratio), fT4, fT3, TSH, triglycerides and cholesterol. Most of these measurements have been conducted on every few weeks during the course of the treatment protocol, but baseline and end-of-treatment values were considered for analysis.

### 3.6. Resource Use

The costs observed included direct inpatient health care costs, outpatient drug acquisition costs (common dominant outpatient portion of eastern European cost matrix of leading chronic disorders), and indirect lost productivity related costs. All patients’ visits to the attending physician, either specialist (infectologist, hepatologist, psychiatrist, nephrologist, etc.) or general practitioner at the primary care unit were evidenced. The authors had full insight into prescribed medicines for home use and have assessed compliance level and real expected consumption (out of pocket expense mostly). Besides aforementioned, number of absenteeism weeks from paid work was evidenced (19).

### 3.7. Costs

To provide a comprehensive insight into real world health care expenditure related to hepatitis C novel treatment, societal perspective was taken into account. The financial value of medical care goods and services consumed was calculated based on the Republican Health Insurance Institute official pricelists for respective years when services were provided, i.e. between 2007 and 2010. It is the only core state owned fund, in charge of most public and private health care funding in Serbia. Based on the bottom-up approach, costs for personnel, drugs needed, overall tertiary medical care, psychiatric support to opioid addicts, nephrologist’s consultations for few patients with renal failure, consumables and all other direct medical care expenses were precisely calculated. Most of the data were obtained from the discharge invoices and the patient files related to the hospital admission and outpatient controls. The Friction Cost method was applied to determine the costs for absenteeism from paid work. This estimate relied on the Serbian Statistics Institute official data per year observed, on mean income of domestic population, taking into account demographic features and education level of an employee (21). Table 1 provides detailed pricelist for year 2010.

**Table 1.** Official Pricelist of Republican Health Insurance Institute of Serbia Upon Which Healthcare Resources Were Valued (Year 2010)

Official pricelist of Main Resources Consumed	Euro
**Drug acquisition**	
Pegasys 180	170.68
Pegasys 135	144.68
Copegus	579.84
**Secondary health care services**	
Infusion drug administration	2.45
Obtaining venous blood sample	2.30
Infectious diseases clinic hospital admission charge per day	15.38
Intensive care hospital per day	12.22
Complete blood count determination	2.73
Determination of antitissueantibodies using immunofluorescence method	16.93
Elisa test for HTLV III (HIV)	46.97
Determination of total antibodies to C antigen of HB virus using EIA method	16.29
Puncture of the liver	7.81
Ex tempore biopsy of tissue with elaboration of permanent preparation	10.47
Highly efficient dialysis	26.55
**Indirect lost productivity costs**	
Official average gross nominal salary per year in Serbia 2007	5,257.03
Official average gross nominal salary per year in Serbia 2008	5,505.26
Official average gross nominal salary per year in Serbia 2009	5,553.26
Official average gross nominal salary per year in Serbia 2010	6,237.31

### 3.8. Analysis

Statistical analyses were performed with Statistica, version 10 (StatSoft Inc, Tulsa, OK, USA). Normality of data distribution was assessed by Shapiro-Wilk test. When the distributions of costs (overall direct and indirect) were non-normal, data were log-transformed to normality or the median, and interquartile range was used as a measure of central tendency and dispersion, respectively. The effects of sex, risk factors, comorbidity, and stage of disease and HCV genotype on cost were evaluated using Kruskal-Wallis analysis of variance by ranks. The correlation of age, viral load and the length of hospitalization with cost were tested using Student t-test and Spearman's rank correlation coefficient for normally and non-normally distributed data, respectively. Comparison of viral load before and after treatment was performed using the repeated measures ANOVA. For all statistical procedures, the difference was considered significant at the level of P < 0.05. The economic assessment was performed based on the intention-to-treat principle. The mean differences in direct medical, indirect, and total costs between both groups and confidence intervals were provided. The effects difference should have been confirmed by multiple analytical measurements of key biochemical variables indicating liver function (RNA viral load, bilirubin, transaminase enzymes, alfa fetoprotein, albumins). Incremental cost-effectiveness ratios (ICERs) were calculated as the difference in total costs between the two groups divided by the difference in effects for viral RNA decrease induced by therapy (22). The probability of PEG INF-α2a plus RBV treatment being more cost-effective among patients with viral genotype 2 or 3 infection as compared to those with genotype 1 or 4 infection was presented as scatter plot.

## 4. Results

### 4.1. Patients

Thirty men and 51 women, with a median age of 40 years (range 19–75 years) completed the study. Of those, 53 were infected by CHC virus genotype 1 or 4 (genotype group I), and 28 had CHC virus genotype 2 or 3 infection (genotype group II). In addition, 12 and 11 subjects were diagnosed with chronic renal failure and mental and behavioral disorders due to abuse of opioids, respectively. Patient compliance was unusually high because of low affordability of interferon treatment for hepatitis C infection in upper-middle income Serbia (23). As the drug cost was not charged to the patients, all of them completed the treatment according to the protocol, and only one patient was lost to follow up before the end of the trial. A single case of sudden and unpredictable severe drug-induced adverse event of anaphylactic reaction was recorded, therefore further participation of the patient was terminated. See Table 2 for detailed clinical characteristics of the population sample observed.

**Table 2.** Patient’s Clinical Data Outsourcing From Attending Infectologist’s Ordinary Follow Up Process

	Genotypes 1, 4	Genotypes 2, 3	All
**Number of patients**	53	28	81
**Age, average**	43.19	39.96	42.07
**Sex**			
Male	34	17	51
Female	19	11	30
**Number of “significant” comorbidities – (Opioid addiction and renal failure** **- (absolute number - mean value within the group)**	1.25	1.25	1.25
**Stage of disease 1-4 (mild-severe) - according to liver biopsypathohistology** **(absolute number - mean value within the group)**	1.89	2.46	2.11
**Risk factors presence – Intravenous psychoactive substance abuse history(absolute number of patients possessing this factor)**	7	8	15
**Risk factors presence – Blood transfusion received before 1993(absolute number of patients possessing this factor)**	7	3	10
**Risk factors presence – Undergoing hemodialysis in distant past(absolute number of patients possessing this factor)**	2	0	2
**AST, U/L**	102.62	113.61	106.42
**ALT, U/L**	147.06	144.82	146.28
**Bilirubin conjugated,umol/L**	2.65	2.52	2.61
**Bilirubin unconjugated,umol/L**	11.26	10.24	10.91
**AFP,ng/ml**	81.04	69.29	76.77
**Viral RNA load in 1000 copies/µL, baseline**	4,856,553.64	8,562,956.57	6137779.35
**Viral RNA load in 1000 copies/µL, consecutive**	2,042,555.34	1,850,891.43	1976301.15
**Viral RNA Decrease – effectiveness, expressed in 1000 copies/µL**	2,821.91	6,649.31	4161.48
**Blood Proteins value expressed in g/ L**	73.57	72.04	73.02
**Albumins value expressed in g/ L**	45.14	45.43	45.24
**Globulins value expressed in g/ L**	28.46	27.33	28.05
**International Normalized Ratio**	96.76	141.95	115.79
**N of Erythrocytes expressed in 10^12^/ L, baseline**	3.54	3.81	3.63
**N of Erythrocytes expressed in 10^12^/ L, consecutive**	3.12	3.45	3.21
**N of Leucocytes expressed in 10^9^/ L, baseline**	3.38	3.37	3.38
**N of Leucocytes expressed in 10^9^/ L, consecutive**	2.77	3.00	2.86
**N of Granulocytes expressed in 10^9^/ L, baseline**	1.35	1.30	1.33
**N of Granulocytes expressed in 10^9^/ L, consecutive**	1.15	1.14	1.15
**N of Platelets expressed in 10^9^/ L, baseline**	133.20	106.40	124.15
**N of Platelets expressed in 10^9^/ L, consecutive**	94.94	100.48	97.14
**Hemoglobin value expressed in g/ L, baseline**	108.52	117.33	111.37
**Hemoglobin value expressed in g/ L, consecutive (after treatment introduction)**	94.21	104.27	96.98
**fT4,pg/ml**	11.30	11.44	11.35
**fT3,pg/ml**	2.79	2.96	2.85
**Thyroid Stimulating Hormone,microU/ml**	27.21	1.69	18.38
**Triglycerides,mmol/l**	1.37	81.55	1.32
**Cholesterol,mmol/l**	4.49	1.24	4.29

### 4.2. Resource Use Because of INF Treated Hepatitis C

**Figure 1.**
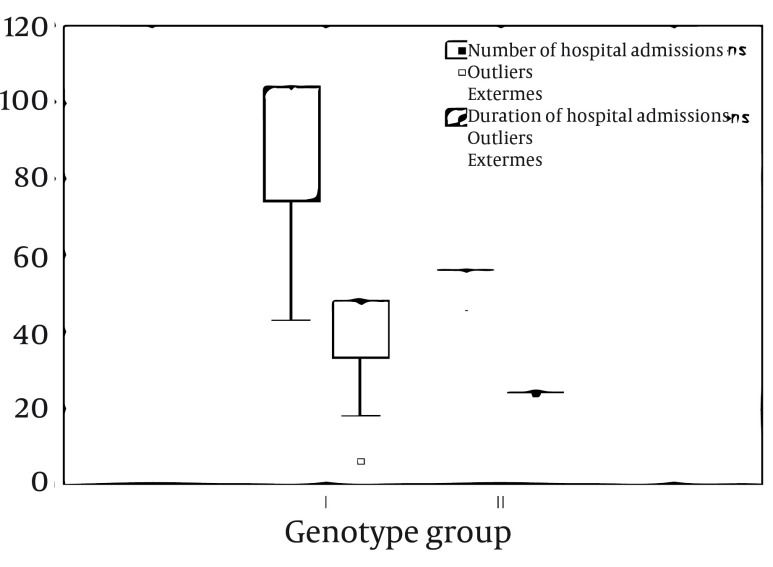
Genotype Determined Differences Between Number and Duration of Hospital Admissions (Genotype Group I: Genotype 1 and 4; Genotype Group II: Genotypes 2 and 3)

The mean number of hospital admission per patient (most of them only 1-2 days, drugs administration and an overnight stay for safety reasons) was 41.60 +/- 10.90 for genotype group I and 23.50 +/- 1.49 for group II. Total duration of hospital admissions was on average 92.47 +/- 19.49 for group I and 54.96 +/ - 3.07 for group II. Moreover, visits to medical facilities were more often (R = 0.72, P < 0.001), while at the same time duration of hospital admission was longer (R = 0.75, P < 0.001), among patients with virus genotype 1 or 4 (Figure 1).

### 4.3. Costs Because of INF Treated Hepatitis C

An in-depth evidence of direct medical costs of inpatient care, outpatient controls, drug acquisition costs at home and absenteeism related indirect costs were acquired for 81 patients. Overall direct costs were significantly higher (H = 4.25, P = 0.039) in patients infected by CHC virus genotype 1 or 4 (median: 14,357.40, IQR: 8,915.55-20,081.12) compared to those with genotype 2 or 3 (median: 10,782.03, IQR: 8,596.72-13,348.28). Similarly, overall indirect cost was significantly higher (H = 54.43, P < 0.001) in genotype group I (median: 921,892.72, IQR: 758,750.31- 155,203.76) compared to genotype group II (median: 2,975.01, IQR: 2,907.94- 3,000.95). Separate comparison within direct costs revealed higher total cost of medical care (R = 0.25, P = 0.024) in first (genotypes 1 or 4) compared to second genotype group (genotypes 2 or 3), while the similar tendency, but not significant difference, was observed for total drug acquisition (t =-1.82, P = 0.072). Although most healthcare related expenses incurred in both groups, were outsourcing from brand peg-Interferon α2a market value itself (24), the total costs (both direct and indirect) were still significantly higher (t =- 4.7, P = 0.035) in patients infected by CHC virus genotype 1 or 4 (mean ± SD: 12,751.55 ± 5,588.06) compared to other genotype group (mean ± SD: 10,580.57 ± 3,973.02). Yet, total cost per hospital day was not significantly different between the groups (t = 0.39, P = 0.700) (Table 3).

**Table 3.** Mean Costs in Different Genotype Groups: Genotype Group I - Genotype 1 or 4, Genotype Group II - Genotype 2 or 3

	Genotype group I		Genotype group II		All	
	Mean	SD	Mean	SD	Mean difference	(95% CI)
**Direct medical costs**	2,191.79	4,178.36	620.31	393.77	1,571.47	(15.60; 3,127.35)
**Nonclassified, (Administrative expenses, Nursing care, Other services e.g. social care, Transport, Patient education, Advisory services, Expert opinion providing)**	867.64	2,6960.32	23.08	46.95	844.56	(-175.77; 1,864.89)
**Hospital Admission, (Specialist’s consultations, Intensive Care Unit admissions and Consumables included)**	736.08	648.69	427.61	342.55	308.46	(46.45; 570.47)
**Laboratory, (Blood Biochemistry,Pathohistology, Cytology examinations, Law medicine, Forensic services)**	129.98	106.58	112.51	85.46	17.47	(-28.88; 63.83)
**Imaging techniques, (radiology and nuclear medicine diagnostics)**	92.05	85.66	75.28	63.89	16.77	(-19.87; 53.42)
**Interventions, (Surgical Interventions, interventional radiology, Radiation Therapy Procedures, Psychotherapy, Dialysis,Physiatrictreatment)**	366.03	1,1430.96	4.81	7.22	361.22	(-71.64; 794.09)
**Drug acquisition, (inpatient and outpatient consumption)**	12,751.55	5,5880.06	10,580.57	3,973.02	2,170.98	(-161.62; 4,503.59)
**Indirect costs, (lost productivity)**	1,158,194.76	561,145.28	2,991.94	183.68	1,155,202.82	(946,732.09; 1,363,673.552)
**Total costs, per patient per treatment course**	20,179.68	5,9070.26	14,192.82	4,040.64	5,986.86	(3,540.16; 8,433.55)

### 4.4. Effects

Among all participants, 50 patients with CHC were infected by HCV genotype 1, one patient with HCV genotype 2, 27 patients with HCV genotype 3, and 3 patients with HCV genotype 4. Several key surrogate markers of liver function were observed as clinical endpoints, but no statistically significant differences were found for ALT (P = 0.910), AST (P = 0.454), conjugated (P = 0.686) or unconjugated bilirubin (P = 0.203), as well as regarding viral RNA load before the treatment (P = 0.422), after the treatment (P = 0.766), and viral RNA load decrease (P = 0.863). Upon treatment protocol completion, SVR was detected in 87 % in genotype group I and 86 % in genotype group II, and this difference obviously, was not statistically significant. (Table 4) In addition, comparison of viral load before and after treatment revealed significant decrease in both genotype groups I (F = 7.65, P = 0.002) and II (F = 22.40, P < 0.001).

**Table 4.** Mean RNA Load in Different Genotype Groups: Genotype Group I - Genotype 1 or 4, Genotype Group II - Genotype 2 or 3

	Genotype group I		Genotype group II		Both groups	
	Mean	SD	Mean	SD	Mean difference	(95% CI)
**Viral RNA load (before treatment)**	4,856,553.64	5,249,964.56	8,562,956.57	1,.620,200.28	-3,706,402.93	(-7,841,429.79; 28,623.93)
**Viral RNA load (after treatment)**	2,042,555.34	13,799,742.46	1,850,891.43	5,250,044.85	191,663.91	(-5,124,223.93; 5,507,551.75)
**Viral RNA Decrease-effectiveness**	2,829,719.00	13,527,307.58	6,711,992.29	15,063,914.25	-3,882,273.29	(-10,325,713.86; ,561,167.29)

### 4.5. Cost Effectiveness

**Figure 2.**
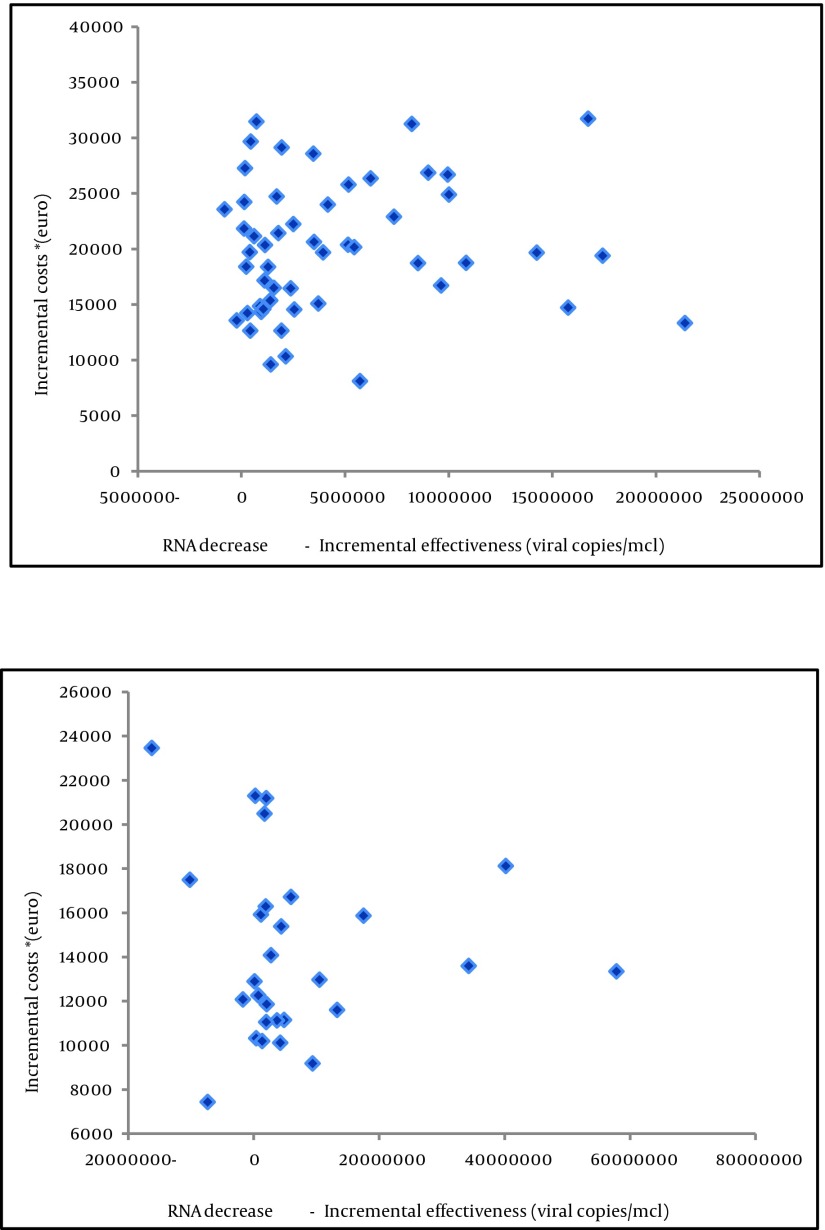
Incremental Cost-Effectiveness Ratio, Genotypes 1, 4 (up) vs. Genotypes 2, 3 (below)

The differences in costs and effects and cost-effectiveness mean values are presented in Tables 2 and 3, and Figure 2. The costs per additional 1000 viral RNA copies/µl decrease of serological remission *(unit of effectiveness) was €18.10 for genotype group I, and just €9.81 for genotype group II. Although patients infected by HCV genotypes 1 and 4 were significantly more expensive to treat, clinical response for viral RNA load decrease was much better among patients with HCV genotypes 2 and 3 infection. The overall association of cost-effectiveness scores between the groups was not significantly different (t = 0.53, P = 0.597). Figure 2 provides an insight into the incremental cost-effectiveness distribution and the pattern of patient’s estimated values grouping. Patients with genotype group I exhibit tendency towards grouping together on the right side of the y axis, although some values are dispersed beneath X axis, most reside almost evenly located above the x axis, in the upper right quadrant (more responsive + more expensive cases). On the other hand, patients with genotype group II exhibit very dispersed pattern, but almost all are grouped in the upper right quadrant of the graph too. An acceptability curve would be a method of choice if an intervention is both more expensive and effective (25). We avoided it here because other figures provide policy makers by straight forward evidence of our results. For further details consult Figure 2.

## 5. Discussion

The core research question posed by this trial was that mean cost-effectiveness value can vary substantially, depending on the genotype of hepatitis C virus that causes infection. It was based on previously well-documented published reports on different clinical course of infection among different HCH viral genotypes (26, 27). We concluded that, patients with HCV genotype 1 or 4 infection imposed significantly higher direct medical costs, excluding drug acquisition ones by comparing cost matrix between the groups. Nevertheless, after adding consumed pegylated interferon alfa plus ribavirin protocol value, we observed major expenses moving towards patients with HCV genotype 2 or 3 infection. Total direct costs, if taking into account nominal gross domestic product parity, were comparable to that of Solomon et al (12). Finally, after adding lost productivity value estimates for our patients, we concluded that total mean costs, observed either per patient or per hospital admission day, are even 25 % higher among patients with genotype group II. Our observations regarding stage of disease and comorbidities impact to patterns of health services utilization and overall costs were mostly in line with the findings of Wong et al. (13). Authors observed statistically significant differences between groups for few clinical outcomes too, such as AST enzyme and bilirubin, both widely accepted surrogate markers of chronic hepatitis activity (28). Particularly evident difference was noticed for viral RNA decrease level in favor of the genotype group II, contingent with the findings of Singal et al. (29). The observed serum viral load decrease was more than twice as extensive as in the genotype group II, which can be explained by 1 and 4 virus epidemiology and infection prognosis (27). Based on significant differences in total costs and effectiveness, we calculated that among patients with HCV genotype 2 or 3 infection PEG-INF-α plus RBV treatment tended to be more cost-effective than those with HCV genotype 1 or 4 infection, but the difference was not statistically significant. The dosing regimen of pharmacological intervention assessed was to some extent more intensive in patients with genotype group II, as recommended by widely accepted guidelines (17). The same group had significantly more often visits to medical facilities and mean duration of hospital admissions. Although the duration of treatment on average was shorter in this group, this effect was not considered as potential bias, as all calculations were performed at individual patient level. The source of this small difference in the amount of hospital resources consumed can be explained by respectively longer duration of treatment protocol in genotype group I. These patients simply had more time to resolve any ongoing treatment related health difficulties, because they were seeing their attending physician for almost a year. Assessed level of compliance was similar between the groups and very high in general. An average patient in both groups incurred even €18,121.04 costs during protocol duration of less than a year. Pegylated interferon alpha 2a and ribavirin were provided to the patients free of charge and donated by Rosche® Serbia without clinical trial related limitations. As such an expensive treatment could not be reimbursed officially in upper-middle income Serbia (21), the reason for the high patients’ compliance could be explained by otherwise seldom affordability of the best CHC treatment (30). Although the patients groups in this study were fairly similar not only in size, but also in demographic structure and medical background (age sex, disease stage, concomitant morbidities), thus minimizing the bias. Only one patient did not complete the trial due to severe drug adverse reaction. Few other patients had some minor clinical endpoints missing, but these files were properly included in overall picture (31). Because of the common skewed distribution of the cost data (9), these kinds of trials usually demand large sample size. Unfortunately, the present study was constrained by financial feasibility of big scale follow-up without industrial funding. While the authors were truly dedicated to precise and responsible clinical follow up and costs evidence, but the key limitation of our trial was the limited number of participants. On the other hand, among strengths of this research we would like to point out to decent robustness of results. The mean medical care costs of chronic hepatitis C could be compared with the results previously reported by Solomon, Wong, and Garcia (10, 12, 13). All of aforementioned studies, although methodologically comparable with our trial, were conducted in high-income economy clinical settings. Direct medical costs were higher in the first as compared to the second genotype group, mostly due to drug acquisition and indirect lost productivity costs. Few foreign trials reported substantially higher absenteeism from paid work costs (32). We tend to explain this difference by significantly higher labor wages in high income markets (33). Standard PEG-INF-α plus RBV based pharmacological protocol seems to be more cost-effective intervention for chronic hepatitis C infection treatment among HCV genotype 2 or 3 as compared to patients with HCV genotype 1 or 4 infection. This may be related to the nature of infection itself, as the latter group is considered prognostically better and less demanding (29). Policy makers should strive to sustain absenteeism closer to the bottom line possible, taking into account inevitably high drug acquisition costs of immunobiological treatment. In such circumstances, this intervention should prove even more cost-effective among patients infected by prognostically more severe viral genotypes. Results outsourcing from this trial should be interpreted generally affirmative for future reimbursement of this intervention. Our main conclusion is that policy makers should consider willingness to pay threshold differentially, depending on HCV viral genotype detected in patients.

## References

[1] Strader DB, Wright T, Thomas DL, Seeff LB (2004). Diagnosis, management, and treatment of hepatitis C.. Hepatology..

[2] Pecic V, Stankovic-Djordjevic D, Nestorovic M, Radojkovic M, Marjanovic H, Ilic B (2011). Hepatitis C virus-related hepatocellular carcinoma and liver cirrhosis.. J Buon..

[3] Chevaliez S, Pawlotsky JM (2007). Hepatitis C virus: virology, diagnosis and management of antiviral therapy.. World J Gastroenterol..

[4] De Francesco R, Migliaccio G (2005). Challenges and successes in developing new therapies for hepatitis C.. Nature..

[5] Le Guillou-Guillemette H, Vallet S, Gaudy-Graffin C, Payan C, Pivert A, Goudeau A (2007). Genetic diversity of the hepatitis C virus: impact and issues in the antiviral therapy.. World J Gastroenterol..

[6] Lee CM, Hung CH, Lu SN, Changchien CS (2008). Hepatitis C virus genotypes: clinical relevance and therapeutic implications.. Chang Gung Med J..

[7] Zein NN (2000). Clinical significance of hepatitis C virus genotypes.. Clin Microbiol Rev..

[8] Kuljic-Kapulica N, Jovanovic D, Savic D, Ristanovic E, Nozic D, Rajic R (2010). [Therapy of chronic hepatitis C--virologic response monitoring].. Vojnosanit Pregl..

[9] Briggs A, Shiell A (1996). Interferon-alpha in hepatitis C. Dosage, costs and benefits.. Pharmacoeconomics..

[10] Garcia de Ancos JL, Roberts JA, Dusheiko GM (1990). An economic evaluation of the costs of alpha-interferon treatment of chronic active hepatitis due to hepatitis B or C virus.. J Hepatol..

[11] Hartwell D, Jones J, Baxter L, Shepherd J (2011). Peginterferon alfa and ribavirin for chronic hepatitis C in patients eligible for shortened treatment, re-treatment or in HCV/HIV co-infection: a systematic review and economic evaluation.. Health Technol Assess..

[12] Solomon M, Bonafede M, Pan K, Wilson K, Beam C, Chakravarti P (2011). Direct medical care costs among pegylated interferon plus ribavirin-treated and untreated chronic hepatitis C patients.. Dig Dis Sci..

[13] Wong JB (1998). Interferon treatment for chronic hepatitis B or C infection: costs and effectiveness.. Acta Gastroenterol Belg..

[14] Nikolic P, Jemuovic L, Delic D, Korac M, Boricic I (2000). Treatment of chronic hepatitis C with interferon alpha – our results.. Acta Infectologica Yugoslavica..

[15] Simmonds P, Bukh J, Combet C, Deleage G, Enomoto N, Feinstone S (2005). Consensus proposals for a unified system of nomenclature of hepatitis C virus genotypes.. Hepatology..

[16] Faul F, Erdfelder E, Lang AG, Buchner A (2007). G*Power 3: a flexible statistical power analysis program for the social, behavioral, and biomedical sciences.. Behav Res Methods..

[17] Berg T, von Wagner M, Nasser S, Sarrazin C, Heintges T, Gerlach T (2006). Extended treatment duration for hepatitis C virus type 1: comparing 48 versus 72 weeks of peginterferon-alfa-2a plus ribavirin.. Gastroenterology..

[18] Maruoka D, Imazeki F, Arai M, Kanda T, Fujiwara K, Yokosuka O (2012). Longitudinal changes of the laboratory data of chronic hepatitis C patients with sustained virological response on long-term follow-up.. J Viral Hepat..

[19] Jacobs P, Fassbender K (1998). The measurement of indirect costs in the health economics evaluation literature. A review.. Int J Technol Assess Health Care..

[20] Matsuura K, Tanaka Y, Hasegawa I, Ohno T, Tokuda H, Kurbanov F (2009). Abbott RealTime hepatitis C virus (HCV) and Roche Cobas AmpliPrep/Cobas TaqMan HCV assays for prediction of sustained virological response to pegylated interferon and ribavirin in chronic hepatitis C patients.. J Clin Microbiol..

[21] Serbia IoPHo. (2010). Health statistical yearbook of Republic of Serbia 2010..

[22] Annemans L (2008). Health economics for non-economists: an introduction to the concepts, methods and pitfalls of health economic evaluations..

[23] Gajic-Stevanovic M, Vuksa A, Zivkovic S, Teodorovic N (2010). Cost of primary health care in the Republic of Serbia for the period 2006-2008.. Serbian Dental J..

[24] Gerkens S, Nechelput M, Annemans L, Peraux B, Mouchart M, Beguin C (2007). A health economic model to assess the cost-effectiveness of PEG IFN alpha-2a and ribavirin in patients with mild chronic hepatitis C.. J Viral Hepat..

[25] O'Hagan A, Stevens JW, Montmartin J (2000). Inference for the cost-effectiveness acceptability curve and cost-effectiveness ratio.. Pharmacoeconomics..

[26] Carlsson T, Quist A, Weiland O (2008). Rapid viral response and treatment outcome in genotype 2 and 3 chronic hepatitis C: comparison between two HCV RNA quantitation methods.. J Med Virol..

[27] Marcellin P, Reau N, Ferenci P, Hadziyannis S, Messinger D, Tatsch F (2012). Refined prediction of week 12 response and SVR based on week 4 response in HCV genotype 1 patients treated with peginterferon alfa-2a (40KD) and ribavirin.. J Hepatol..

[28] Romeo JM, Ulrich PP, Busch MP, Vyas GN (1993). Analysis of hepatitis C virus RNA prevalence and surrogate markers of infection among seropositive voluntary blood donors.. Hepatology..

[29] Singal AK, Anand BS (2010). Tailoring treatment duration to 12 to 16 weeks in hepatitis C genotype 2 or 3 with rapid virologic response: systematic review and meta-analysis of randomized controlled trials.. J Clin Gastroenterol..

[30] Glied S (2009). Mandates and the affordability of health care.. Inquiry..

[31] Noble SM, Hollingworth W, Tilling K (2012). Missing data in trial-based cost-effectiveness analysis: the current state of play.. Health Econ..

[32] Su J, Brook RA, Kleinman NL, Corey-Lisle P (2010). The impact of hepatitis C virus infection on work absence, productivity, and healthcare benefit costs.. Hepatology..

[33] Shah NM, Brieger WR, Peters DH (2011). Can interventions improve health services from informal private providers in low and middle-income countries?: a comprehensive review of the literature.. Health Policy Plan..

